# Development and Application of a Triplex TaqMan Quantitative Real-Time PCR Assay for Simultaneous Detection of Feline Calicivirus, Feline Parvovirus, and Feline Herpesvirus 1

**DOI:** 10.3389/fvets.2021.792322

**Published:** 2022-02-08

**Authors:** Nan Cao, Zhihui Tang, Xiyu Zhang, Wanyan Li, Bingxin Li, Yunbo Tian, Danning Xu

**Affiliations:** ^1^Guangdong Province Key Laboratory of Waterfowl Healthy Breeding, College of Animal Science and Technology, Zhongkai University of Agriculture and Engineering, Guangzhou, China; ^2^MOE Joint International Research Laboratory of Animal Health and Food Safety, College of Veterinary Medicine, Nanjing Agricultural University, Nanjing, China

**Keywords:** triplex qRT-PCR, FCV, FPV, FHV-1, clinical detection

## Abstract

As companion animals, felines play an important role in human's family life, and their healthcare has attracted great attention. Viruses such as feline calicivirus (FCV), feline herpesvirus 1 (FHV-1), and feline parvovirus virus (FPV) are the most common pathogens that cause severe infectious disease in baby cats. Thus, preclinical detection and intervention of these three viruses is an effective means to prevent diseases and minimize their danger condition. In this study, a triplex TaqMan quantitative real-time polymerase chain reaction (qRT-PCR) assay was developed to detect these three viruses simultaneously. The detection limit of FPV, FCV, and FHV-1 was 5 × 10^1^ copies/assay, which exhibited higher sensitivity (about 10- to100-fold) than conventional PCR. The coefficients of variation (CVs) of the intra-assay variability were lower than 1.86%, and that of inter-assay variability were lower than 3.19%, indicating the excellent repeatability and reproducibility of the triplex assay. Additionally, the assay showed good specificity. Finally, samples from 48 cats were analyzed using the established assay and commercial kits. As a result, the total positive rates for these viruses were 70.83 or 62.5%, respectively, which demonstrated that the developed qRT-PCR assay was more accurate than the commercial kits and could be used in clinical diagnosis.

## Introduction

As companion animals, cats play an important role in human's life, and their health care has attracted great attention. However, infectious disease caused by feline calicivirus (FCV), feline herpesvirus 1 (FHV-1), and feline parvovirus virus (FPV) still pose a threat to cats' health (1, 2).

FCV is an RNA virus belonging to *Vesivirus* of Caliciviridae. Studies show that FCV is the most widespread feline virus, especially in multi-cat households and shelters, with overall prevalence ranges from ~15–31% (3). The morbidity of FCV can reach to 90% in some colonies (4, 5). More importantly, the clinically recovered felines may become the virus carriers (5). FHV-1 is a double-stranded and enveloped DNA virus belonging to α-herpesvirinae of Herpesviridae, which is widely distribute in the world. FHV-1 mainly infects felidae, especially the kittens aged 2–3 months. When the kittens are infected with this virus, secondary infections are likely to occur due to the poor immunity caused by FHV-1, and the final mortality rate can reach to 70% (6, 7). Panleukopenia, caused by FPV, is an acute, highly contagious, and, sometimes, fatal feline viral disease, which distribute throughout the world (8). FPV is a DNA virus belonging to *Parvovirus* of *Parvoviridae*. The use of vaccines has greatly reduced the incidence of these three viruses, but in some rural areas of developing countries, the three viruses are still highly prevalent, and cases of mixed infection are often reported (especially in multi-cat households and stray catteries) (9). In the early stages of infections, similar clinical symptoms of the three diseases, such as depression, anorexia, sneezing, diarrhea, conjunctival congestion, eye–nose secretion, and dyspnea (2, 10–13) make it difficult to identify the virus with the naked eye, which could result in the missing of proper treatment time. Moreover, when selecting cats as experimental animals for scientific research, these three viruses must be detected and excluded. Therefore, it is important to establish a suitable method for the differential detection of these three viruses. The current diagnostic methods for these diseases include serological testing (14, 15), virus isolation, and identification (16, 17), polymerase chain reaction (18–20). As we know, the serological diagnosis could give false-positive results in some time, and virus isolation have not been widely used in clinical diagnosis because of high costs and being time consuming. Therefore, establishment of a time-saving, labor-saving, sensitive, and efficient detection method is urgently needed.s

The PCR method has been widely used in the diagnosis of various pathogens because of the specificity, sensitivity, and efficiency. Compared with uniplex PCR, the multiplex PCR greatly improves the detection efficiency by simultaneously amplifying multiple templates in a single reaction (21, 22). Nowadays, a second-generation PCR technology, quantitative real-time PCR (qRT-PCR), has been widely used in the field of scientific research and clinical detection due to its high specificity, sensitivity, and time saving (23–26). Although several PCR methods have been reported for the detection of these three viruses (27–30), most of them have low sensitivity or tedious operation, and no method can be used to detect all three viruses quickly and efficiently at the same time. In this study, a TaqMan triplex qRT-PCR assay (triplex assay) for the simultaneous determination of FCV, FHV-1, and FPV was developed. The method can differentiate these viruses with high sensitivity, specificity, and reproducibility.

## Materials and Methods

### Pathogen and Clinical Samples

FCV (attenuated vaccine strain F9), FHV-1 (attenuated vaccine strain G2620A), and FPV (attenuated vaccine strain MW-1) were purchased from Intervet International B.V. Wild strains of these three viruses were isolated and stored by our laboratory, and the virus titers were 10^8.0^ TCID_50_/0.1 ml for FCV, 10^7.0^ TCID_50_/0.1 ml for FPV, and 10^8.3^ TCID_50_/0.1 ml for FHV-1. The nucleic acids of rabies virus (RV, attenuated vaccine strain Pasteur RIV, purchased from Intervet International B.V.), pseudorabies virus (PRV, attenuated vaccine strain HB-98, purchased from Wuhan Keqian Biology), feline coronavirus (FCoV, positive clinical samples), and feline immunodeficiency virus (FIV, positive clinical samples) were used for specificity test. The complete genome of feline leukemia virus (FeLV, AF052723) was synthesized by Sangon Biotech (Shanghai, China) and used for specificity test, too.

A number of 48 clinical samples (each sample was the mixture of oral swab and rectal swab of one cat) from three animal hospitals in Nanjing were collected from February 2019 to December 2019. All samples were divided into two parts, one for the triplex assay, and the other for commercial kits. At the same time, 15 negative samples (each sample was the mixture of oral swab and rectal swab of one cat), confirmed to be free of FCV, FHV-1, and FPV (27, 31, 32), were used in the study. Methods for sample collection and storage were as described (33).

### Primers and Probes Design

The published VP2 gene of FPV, ORF2 gene of FCV, and TK gene of FHV-1 were obtained from GenBank and aligned by DNAMAN (LynnonBiosoft, USA) to find the conservative regions. Six pairs of specific primers and three specific probes were designed. The specificity of primers was confirmed using BLAST in NCBI before use. Three pairs of longer fragments were used to construct plasmids, and three pairs of shorter fragments and probes (involved in longer fragments) were used for fluorescence detection of three viruses. The three probes were labeled with FAM/BHQ1 (FPV), VIC/BHQ1 (FCV), and Texas Red /BHQ2 (FHV) at its 5′ and 3′ terminals, respectively. The primers and probes were synthesized by Sangon Biotech (Shanghai, China); the details of these oligos are shown in Table 1.

**Table 1:** Primers and probes.

**Name^a^**	**Sequence (5^′^-3^′^)^b^**	**Target** **genes**	**Amplicon** **size (bp)**
FPV-F	CGGGGGTGGTGGTGGTT	VP2	112 bp
FPV-R	GCTTGAGTTTGCTGTGATTTCC		
FPV-P	**FAM** - CTGGGGGTGTGGGGATTTCTACG - **BHQ1**		
FCV-F	CGCCCTACACTGTGATGTG	OFR2	165 bp
FCV-R	GAGTTCTGGGTAGCAACACAT		
FCV -P	**VIC** - ATGTGCTCAACCTGCGCTAACGTGC - **BHQ-1**		
FHV-1-F	ATTTGCCGCACCATACCT	TK	140 bp
FHV-1-R	GCGAGTGGGAAACAGACC		
FHV-1-P	**Texas Red** - CTTTTACATTCCAGACTATCCACAATAACAGG - **BHQ-2**		

a *F, forward primer; R, reverse primer; P, TaqMan probe*.

b *FAM, 6-carboxy-fluorescein; VIC, 6-carboxy-rhodamine; BHQ, black hole quencher*.

### Nucleic Acids Extraction and Standard Plasmid Preparation

The RNA of FCV and the DNA of FHV-1 and FPV were extracted using commercial kits (Sangon Biotech, China). The reverse transcription reaction was performed to synthesize the first-strand cDNA of FCV following the manufacturer's instructions (Thermo Scientific, USA). The concentration and purity of the nucleic acid were determined by the measurement of the absorbance at 260 and 280 nm with a NanoDrop2000 spectrophotometer (Thermo Scientific, USA). All products were stored at −80°C for use.

With the obtained DNA/cDNA as template, FPV, FHV-1, and FCV gene fragments were amplified by PCR. The PCR products were purified and retrieved using a DNA Gel Extraction Kit (Axygen, China). Retrieved fragments were cloned to pMD18T vector (TaKaRa, China) to construct three positive plasmids named pMD18T-FCV, pMD18T-FHV, and pMD18T-FPV, respectively. The positive plasmids were used to establish standard curves. The concentration of plasmids was calculated according to the absorbance measurement, and the way its conversion to the copy number of the plasmid has been described in the previous study (34).

### Experimental Design and qRT-PCR

To obtain a more sensitive, stable, and easy-to-use PCR method, the annealing temperature, primers concentration, and probes concentration for each target gene were carefully optimized. D-optimal design (MODDE 12.1 software) was carried out to comprehensively analyze the influence of these factors (34). The experimental conditions with the highest fluorescence signal and the lowest cycle threshold (Ct) value were designated as the optimal reaction conditions.

The triplex assay was carried out in a final volume of 20.0 μL on LightCycler 96 system (Roche, Switzerland). The composition of the reaction mixture included 10.0 μL of Hieff Unicon^®^ qPCR TaqMan Probe Master Mix (Yeasen Biotech Co., Ltd.), 1.0 μL of template, 0.5 μM of each primer, 0.1 μM of the FPV probe, 0.05 μM of the FCV probe, 0.05 μM of the FHV-1 probe, and 3.6 μL of double-distilled water (ddH_2_O). The thermocycling conditions included 95°C for 2 min, 40 cycles of 95°C for 10 s, and 54°C for 30 s. The uniplex assays were conducted with the thermocycling conditions same as the triplex assay. The standard plasmid pMD18T-FCV, pMD18T-FHV, and pMD18T-FPV served as the positive control while ddH_2_O as the negative control.

### Conventional PCR

Conventional PCR was performed with a standard program in 20.0 μL reaction volume on a PCR cycler (Eppendorf, Germany). The composition of the reaction mixture contained 10.0 μL of 2 × Hieff™ PCR Master Mix (Yeasen Biotech Co., Ltd.), 1.0 μL of template, 0.5 μM of each primer, and 8 μL of ddH_2_O. Cycle times were as follows: 95°C for 5 min (initial denaturation), 40 cycles of 95°C for 30 s (denaturation), 54°C for 30 s (annealing), and 72°C for 20 s (extension), followed by a final step of 10 min at 72°C (extension). The PCR products were detected by 1.5% agarose gel electrophoresis. Three standard plasmids were used as positive control, and ddH_2_O played the role of the negative control.

### Validation of the qRT-PCR Assay

The specificity of the established qRT-PCR assay was confirmed using RV, PRV, FCoV, FIV, and FeLV. To evaluate the sensitivity of the method, the plasmids containing target genes were diluted by 10 times gradient (from 5 × 10^7^ copies/assay to 5 × 10^0^ copies/assay) and subjected to the sensitivity of the triplex assay. The amplification efficiency (AE) and correlation coefficient (R^2^) were used as parameters to evaluate the sensitivity of the triplex assay (35, 36). The repeatability of the method was tested using plasmid as the templates at 5 × 10^7^, 5 × 10^5^, and 5 × 10^3^ copies/assay. Each independent experiment was carried out in triplicate for the intra-assay repeatability test, and triplicate runs over 3 days were performed by different operators for the inter-assay reproducibility test. The sensitivity and reproducibility tests for the triplex assay were conducted again using nucleic acids extracted from FCV, FPV, and FHV-1 wild strains.

### A Pilot Study of the Triplex Assay

We conducted a co-infection experiment to assess the reliability of the triplex assay. Different combinations and proportions of three virus nucleic acids (DNA/cDNA) were mixed and used for the triplex assay. The established method was also used to analyze 48 clinical samples, and the results were compared with that of commercial kits (Mensall, China).

### Statistical Analysis

Data generation and collection were carried out with LightCycler SW 1.1. Data management, analysis, and graphics generation were performed using Microsoft Excel 2007 (Microsoft, USA) and MODDE 12.1 software (Umetrics, Sweden). Results are presented as mean value ± standard deviation (SD). The intra- and inter-assay variations were calculated from the mean Ct values and expressed as coefficients of variation (CV).

## Results

### Optimization and Establishment of the Triplex Assay

**Figure 1:**
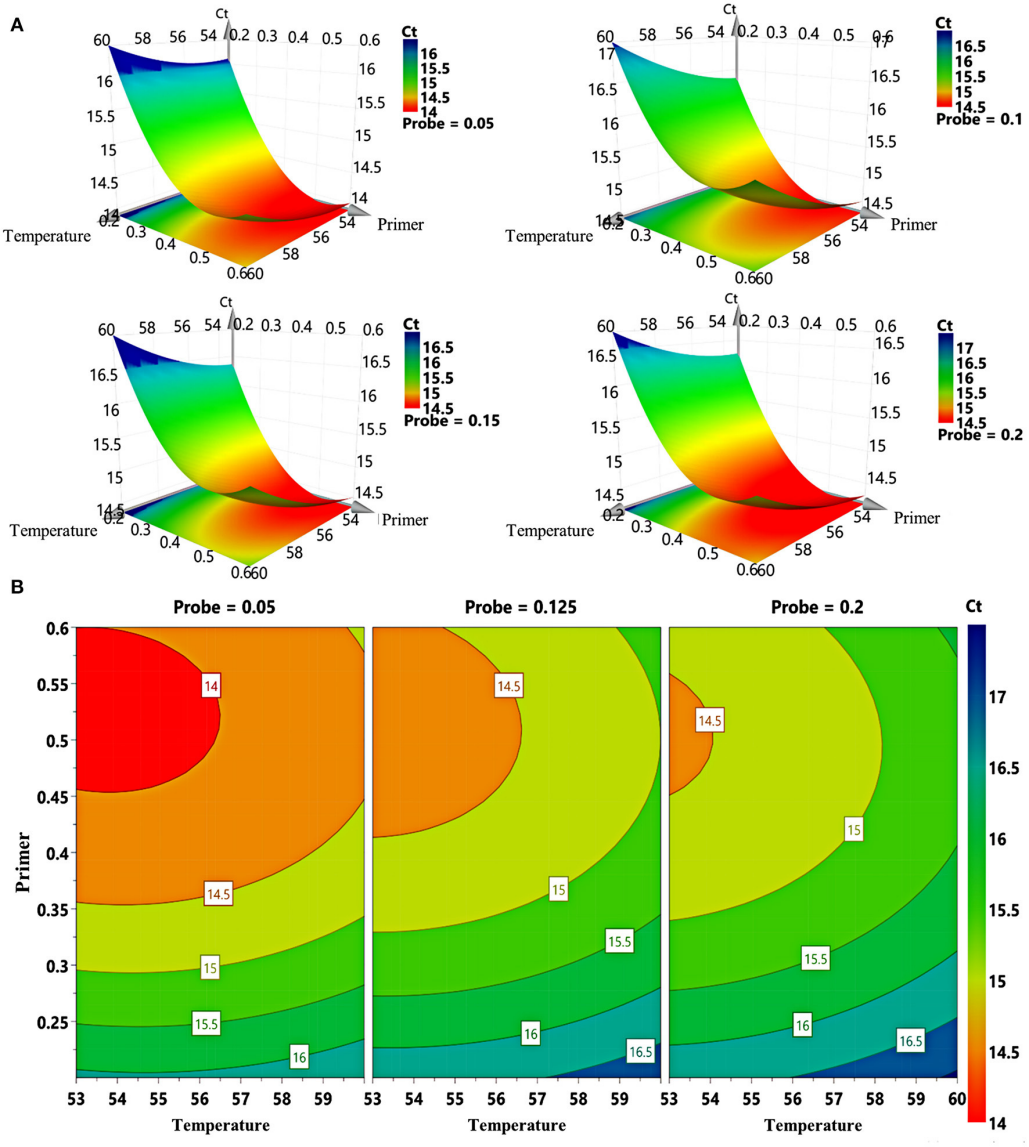
Response surface plots for FCV. **(A)** 3D response graphs based on different combinations of primer concentration, probe concentration, and annealing temperature generated by computer-aided exchange procedure. **(B)** Response surface graphs of primer concentration and annealing temperature at different probe concentrations.

The study adopted D-optimal design consisting of 16 runs to explore the effects of different probe concentrations, primer concentrations, and annealing temperature on Ct values. Taking FCV as example, the three-dimension response surface curves are shown in Figure 1A (data in Supplementary Table S1). Red areas represent lower Ct values, and blue areas represent higher Ct values. The abscissa and ordinate of the lowest point are the optimal conditions. The 4D plots further illustrate the interaction between the three factors (Figure 1B). We find that when the primer concentration is lower than 0.35 μM, the Ct value remains at a high level regardless the changes in probe concentration and annealing temperature. However, when the primer concentration is in the range of 0.4–0.6 μM, the Ct value decreases with a lower probe concentration and annealing temperature.

In addition, we optimized the fluorescence signal of the method (Supplementary Figure S1). The results show that the fluorescence intensities of FCV, FHV-1, and FPV are at high level when the primer concentration is in the range of 0.5–0.6 μM (Supplementary Figures S1A–C). For FPV (Supplementary Figure S1D), the fluorescence intensities of high concentration probes (0.15 and 0.2 μM) is stronger than that of low concentration probes (0.05 μM). However, for FCV (Supplementary Figure S1E) and FHV-1 (Supplementary Figure S1F), a low concentration probe (0.05 μM) can get a stronger fluorescence intensity. Annealing temperature have an obvious effect on fluorescence intensity. As the temperature decrease, the fluorescence intensity increases (Supplementary Figures S1G–I). Finally, as a compromise, the optimized experimental conditions are set as follows: primer concentration at 0.5 μM for each virus, probe concentration at 0.15 μM for FPV, 0.05 μM for FCV or FHV-1, and annealing temperature at 54°C. The cutoff for positivity is determined based on the Ct values of the negative samples, which exceeded 36. Once the Ct value of the sample exceeds 36, it is treated as a negative result.

### Specificity Test

**Figure 2:**
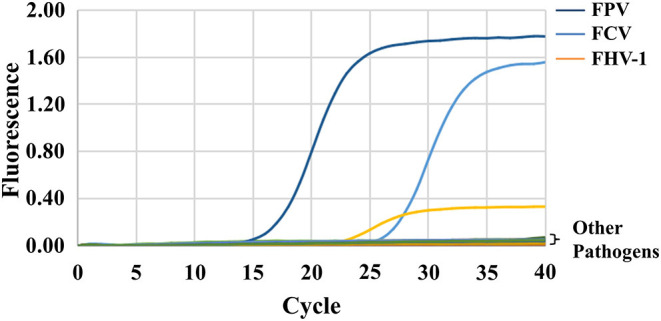
Specificity of the triplex assay. FPV, FCV, FHV-1, and other animal pathogens (including FIV, FeLV, FCoV, RV, and PRV) were selected and tested using the triplex assay. Only FCV, FHV-1, and FPV detected positive.

FPV, FCV, FHV-1, and other animal pathogens (including FIV, FeLV, FCoV, RV, and PRV) were used for the specificity test. The results show that only FCV, FHV-1, and FPV have specific amplification curves, and the Ct values are all <30 (Figure 2). The fluorescence intensities of other pathogens and negative controls are stable at low levels without amplification curves, indicating the high specificity of the triplex assay.

### Sensitivity Test

**Figure 3:**
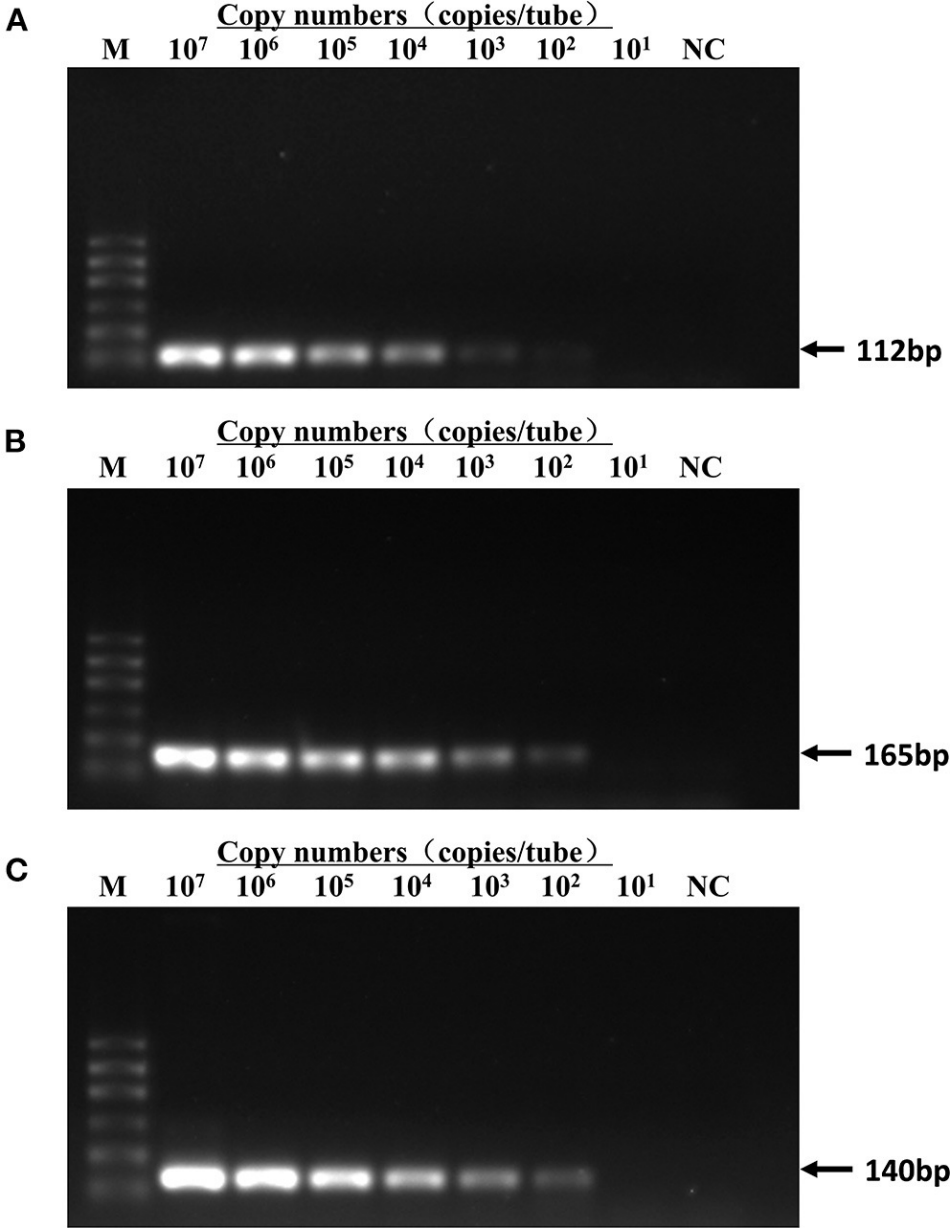
Detection limit of conventional PCR. Templates of pMD18T-FPV **(A)**, pMD18T-FCV-1 **(B)**, and pMD18T-FHV-1 **(C)** were diluted by 10 times gradient to a dilution factor that could not be detected by conventional PCR. The detection limit was 5 × 10^3^ copies/assay for FPV and 5 × 10^2^ copies/assay for FCV and FHV-1. Template amount for curves 2–8 lanes was 5 × 10^7^-5 × 10^1^ copies/assay. M, DL600 marker; NC, negative control.

**Figure 4:**
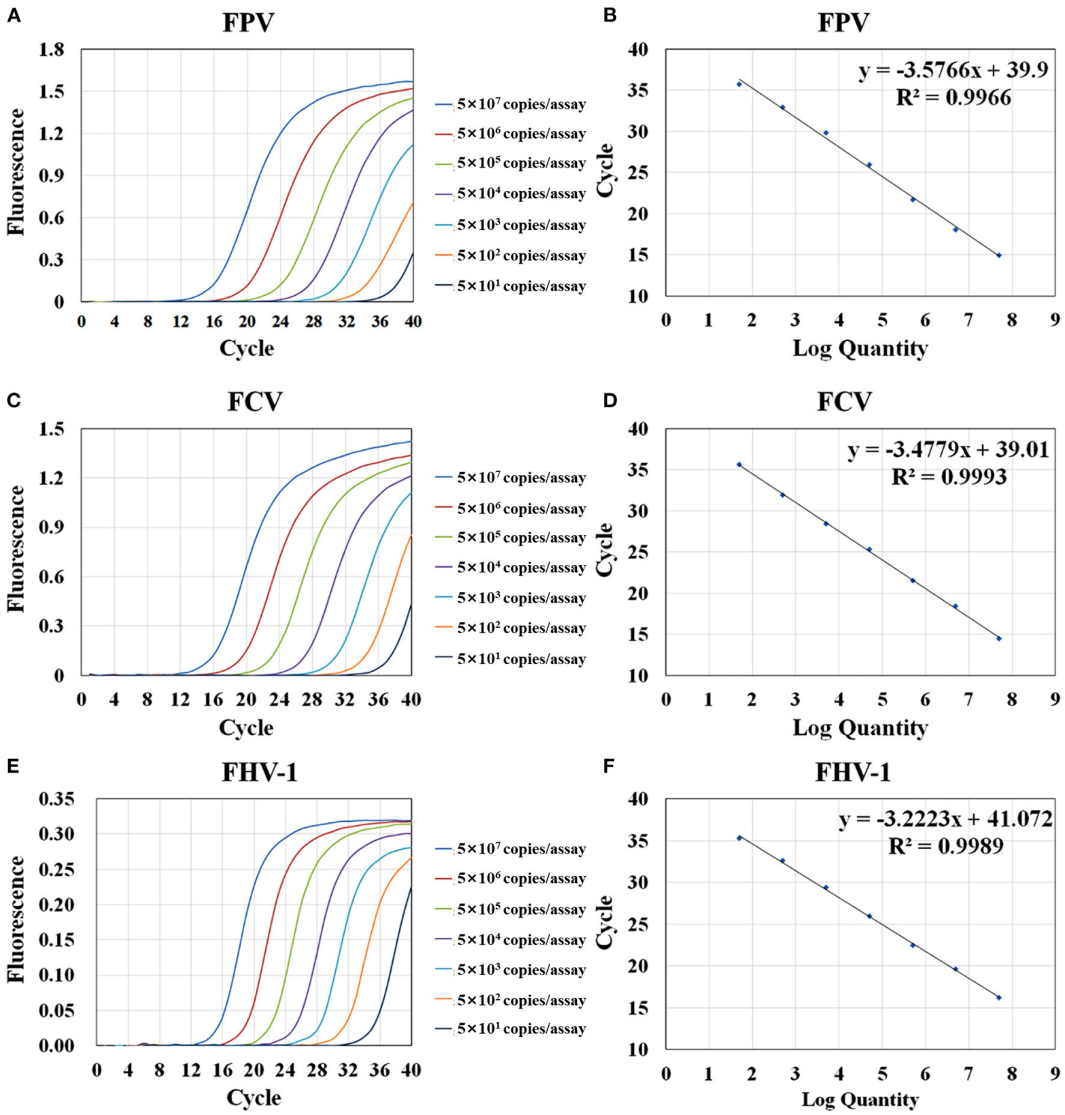
Amplification and standard curves of the triplex assay. Serially diluted plasmids were mixed at equal concentration (from 5 × 10^7^ copies/assay to 5 × 10^1^ copies/assay) and used as template for RT-PCR. Amplification curves of the triplex assay for the detection of FPV **(A)**, FCV **(C)**, and FHV-1 **(E)**. The standard curves of FPV **(B)**, FCV **(D)**, and FHV-1 **(F)** were generated by plotting the Ct values (Y-axis) against the logarithm of copy numbers of plasmids (X-axis).

The sensitivity test of qRT-PCR and conventional PCR was performed with 10- fold serial dilution of plasmids (from 5 × 10^7^ to 5 × 10^1^ copies/assay) and virus nucleic acids (from 10^−1^ to 10^−10^ dilution). The templates of the same dilution were mixed in equal proportions. As shown in Table 2, the Ct value of qRT-PCR remains positive and conforms to the linear trend at 5 × 10^1^ copies/assay for three viruses. However, the sensitivity of conventional PCR is lower. As shown in Figure 3, when FPV is diluted below 5 × 10^3^ copies/assay and FCV and FHV-1 are diluted below 5 × 10^2^ copies/assay, the target fragments are not clearly observed. The standard curves of the triplex assay for these viruses are generated (Figure 4). As shown in the figure, the triplex assay is linear over the range of 5 × 10^7^-5 × 10^1^ copies/assay with an R^2^ value above 0.9966 for all three viruses. Besides, the AE is 90.37% for FPV, 93.88% for FCV, and 104.19% for FHV-1.

**Table 2:** Sensitivity of the real-time PCR.

**Number of** **DNA copies** **(copies/assay)**	**Triplex real-time PCR Ct value^a^** **(mean** **±SD)**			**Uniplex real-time PCR Ct value^b^** **(mean** **±SD)**		
	**FPV**	**FCV**	**FHV-1**	**FPV**	**FCV**	**FHV-1**
5 × 10^7^	14.39 ± 0.25	14.44 ± 0.01	16.17 ± 0.11	14.32 ± 0.25	14.81 ± 0.15	16.11 ± 0.23
5 × 10^6^	18.02 ± 0.45	18.46 ± 0.05	19.62 ± 0.22	18.32 ± 0.05	18.18 ± 0.25	19.69 ± 0.07
5 × 10^5^	21.73 ± 0.16	21.53 ± 0.22	22.47 ± 0.36	21.93 ± 0.02	21.37 ± 0.03	22.82 ± 0.10
5 × 10^4^	25.93 ± 0.06	25.30 ± 0.02	25.98 ± 0.01	25.50 ± 0.66	24.86 ± 0.03	26.28 ± 0.12
5 × 10^3^	29.84 ± 0.13	28.42 ± 0.03	29.40 ± 0.06	27.62 ± 0.16	28.07 ± 0.52	29.95 ± 0.03
5 × 10^2^	32.91 ± 0.21	31.94 ± 0.02	32.61 ± 0.03	31.76 ± 0.35	31.60 ± 0.42	32.98 ± 0.19
5 × 10^1^	35.67 ± 0.83	35.62 ± 0.77	34.93 ± 0.53	34.08 ± 0.33	33.41 ± 0.08	35.25 ± 0.16

a *The result was considered positive if mean Ct value ≤ 36*.

b *Each reaction was performed in triplicate, and the results are shown as the mean ± SD*.

The limit of detection (LOD) was determined by the serial dilutions of the recombinant plasmids that corresponded to the lowest copy number that gave a probability of at least 95% of detecting a PCR positive test result (37, 38). When the plasmids are diluted below 5 × 10^1^ copies/assay, apparently and randomly distributed Ct values in the range of 36–38 are observed in the triplex assay (38, 39). Therefore, we chose 5 × 10^1^ copies/assay as the LOD of the developed method. In the tests using virus nuclei acids as templates, the detection limits of the triplex assay to FPV, FCV, and FHV-1 viruses were 10^−3^, 10^0^, and 10^0^ TCID_50_/assay (Supplementary Figure S3).

We used 5 × 10^1^ copies/assay plasmids as templates for 30 repeated tests. The amplification curves of different viruses are shown in Supplementary Figure S2. The fluorescence intensities of most samples were lower than 0.06. Three samples had amplification curves, and only one sample test FPV positive (Ct value 26.25). Thus, the false positive rate of the triplex assay in FCV and FHV tests was 0 (0/30) and that in FPV test was 3.3% (1/30).

### Repeatability and Reproducibility of the Triplex Assay

In this study, three different concentrations of plasmids were used for the repeatability and reproducibility test of the established qRT-PCR assay. As shown in Table 3, the intra- and inter-assay CVs of Ct values are between 0.68–1.86% and 0.85–3.19%, respectively, indicating that the triplex assay is highly reliable and accurate. The results of the intra- and inter-assay CVs of Ct values using virus nucleic acids as templates are shown in Supplementary Table S2.

**Table 3:** Intra- and inter-assay reproducibility of the triplex assay.

**Name**	**Number of DNA** **copies** **(copies/assay)**	**Intra-assay** ^**a**^			**Inter-assay** ^**a**^		
		**Mean**	**SD**	**CV (%)**	**Mean**	**SD**	**CV (%)**
FPV	5 × 10^7^	14.29	0.15	1.04	14.25	0.21	1.47
	5 × 10^5^	21.66	0.28	1.29	21.98	0.41	1.87
	5 × 10^3^	29.92	0.29	0.96	30.10	0.28	0.93
FCV	5 × 10^7^	14.52	0.10	0.69	14.72	0.40	2.72
	5 × 10^5^	21.42	0.25	1.17	21.28	0.34	1.60
	5 × 10^3^	28.35	0.35	1.23	28.89	0.67	2.32
FHV-1	5 × 10^7^	16.24	0.11	0.68	16.44	0.37	2.25
	5 × 10^5^	22.53	0.42	1.86	22.58	0.72	3.19
	5 × 10^3^	29.34	0.21	0.72	29.58	0.25	0.85

### Co-infection Models and Clinical Sample Detection

As shown in Table 4, the method could detect three viruses at the combination of different concentrations, regardless of triplex or duplex infections, indicating the potential ability of clinical use of the triplex assay.

**Table 4:** Detection of the co-infection models by triplex real-time PCR.

**Co-infection proportion^**a**^**	**Virus titer** **(TCID**_**50**_**/assay)**			**Co-infection real-time PCR Ct value** **(mean** **±SD)**		
	**FPV**	**FCV**	**FHV-1**	**FPV**	**FCV**	**FHV-1**
FPV:FCV:FHV-1 = 10:1:1	10^6^	10^5^	10^5^	9.35 ± 0.11	33.72 ± 0.43	22.87 ± 0.37
FPV:FCV:FHV-1 = 1:10:1	10^5^	10^6^	10^5^	14.09 ± 0.88	29.56 ± 0.52	22.88 ± 0.03
FPV:FCV:FHV-1 = 1:1:10	10^5^	10^5^	10^6^	13.59 ± 0.71	33.88 ± 0.28	19.77 ± 0.56
FPV:FCV = 1:1	10^5^	10^5^	–	14.40 ± 0.47	33.10 ± 0.16	–
FPV:FCV = 10:1	10^6^	10^5^	–	9.69 ± 0.21	34.02 ± 0.12	–
FPV:FHV-1 = 1:1	10^5^	–	10^5^	13.97 ± 0.97	–	22.97 ± 0.16
FPV:FHV-1 = 10:1	10^6^	–	10^5^	9.46 ± 0.24	–	22.30 ± 0.82
FCV:FHV-1 = 1:1	–	10^5^	10^5^	–	33.49 ± 0.39	22.85 ± 0.65
FCV:FHV-1 = 10:1	–	10^6^	10^5^	–	29.52 ± 0.59	22.89 ± 1.06

a *Viruses of different titer were mixed in corresponding proportions and used as templates for real-time PCR*.

Finally, 48 clinical samples were examined using the developed triplex assay (Table 5). The results demonstrated that 34 cats were infected FPV, FCV, or FHV-1. The positive rate for FPV, FCV, and FHV-1 were 29.17% (14/48), 50% (24/48), and 33.33% (16/48), respectively. These samples were also detected by three uniplex commercial kits for comparison. The results show only 30 cats infect these viruses, indicating a relatively lower positive rate of 41.67% (20/48) for FCV and 29.17% (14/48) for FHV-1. It is worth noting that all positive samples detected by commercial kits tested positive by triplex assay. However, four cats were detected FCV positive with the triplex assay, while they were detected negative by the commercial kits. Thus, the established triplex assay shows high accuracy than commercial kits in clinical diagnosis.

**Table 5:** Clinical samples detected by triplex assay and commercial kits.

**Name**	**Triplex assay**		**Commercial kits**	
	**Positive**	**Rate (%)**	**Positive**	**Rate (%)**
FPV	6/48	12.50	6/48	12.50
FCV	8/48	16.67	6/48	12.50
FHV-1	2/48	4.17	2/48	4.17
FPV+FCV	4/48	8.33	4/48	8.33
FPV+FHV-1	2/48	4.17	2/48	4.17
FCV+FHV-1	10/48	20.83	8/48	16.67
FPV+FCV+FHV-1	2/48	4.17	2/48	4.17
Total	34/48	70.83	30/48	62.50

## Discussion

In developed countries, the incidence of feline infectious diseases can be controlled through vaccination and disinfection procedures, while it remains at a higher lever in developing countries. Among these diseases, FCV, FHV-1, and FPV are widely distributed all over the world and greatly endanger the health of felines. The purpose of this article was to establish a triplex assay for the simultaneous detection of these three viruses.

The design of primers and probes is the key step of triplex qRT-PCR development. The interference between different primer and probe pairs should be considered. In this study, through several experiments, we finally screened out the primer and probe pairs with similar melting temperature and few dimers. As we know, the annealing temperature can affect the specificity and amplification efficiency of PCR (40). High annealing temperature will reduce the binding efficiency of primers and templates, and low annealing temperature will lead to a non-specific amplification in the reaction system. In addition, the concentration of primers and probes can also affect the PCR amplification efficiency, while low concentration can lead to incomplete reaction, and high concentration can inhibit the reaction. Therefore, a D-optimal design was adopted in this study to explore the impact of these three factors (annealing temperature and concentration of primers and probes) on the triplex qRT-PCR. As shown in the result, when the primer concentration is lower than 0.35 μM, the Ct value remained high regardless of the change in probe concentration and annealing temperature, which might be a result of incomplete reaction caused by insufficient primers.

Theoretically, the amount of PCR products would double after each cycle. But in fact, the AE of PCR cannot reach 100% due to the influences of different factors such as enzymes, deoxyribonucleotide triphosphates (dNTPs), primers, and templates. Therefore, we generated the standard curves to observe the AE of the triplex assay. In general, the AE is acceptable between 90 and 110%, when high level of AE indicates non-specific amplification or primer dimers formation, and a low level maybe caused by improper experimental conditions or reagent concentration. In our assay, the AEs of the standard curves of the three plasmids are all within this range and follow a good linear trend (R^2^ > 0.99), indicating that the experimental conditions are well designed (41).

The lowest sensitivity of this triplex assay is 5 × 10^1^ copies/assay for all three plasmids, which demonstrates a 10- to 100- fold increase than that of conventional PCR. Specificity test shows that the method can detect each of the three viruses and has no cross-reaction with other selected pathogens. Viruses often infect felines in different combinations and concentrations, especially in stray cats in young age. Therefore, we constructed the co-infection model in this study, and it showed satisfactory accuracy and sensitivity when detecting different combinations of these three viruses.

Finally, the clinical sample analysis showed that the positive rate of FCV and FHV-1 detected by triplex assay was higher than that of the commercial kits. The reason could be that we considered most of the epidemic strains in recent years when designing primers for the assay, but the commercial kits had been developed a few years ago, which might miss the prevalent virus. These results also remind us to update the detection method regularly. In this study, we found that the mixed infections of these viruses were common. Among them, the incidence of FCV and FHV-1 co-infection was the highest, with a ratio of 20.83% (10/48). Most of the co-infection cases were stray cats or kittens within 3 months. This phenomenon can be attributed to that stray cats are usually non-vaccinated and more likely to carry the viruses and spread them to domestic cats. Therefore, our research highlights the great need for virus surveillance in stray cats.

## Conclusions

The TaqMan triplex assay established in this study possesses high specificity, sensitivity, and reproducibility for the detection, quantitation, and differentiation of FPV, FCV, and FHV-1 simultaneously.

## Data Availability Statement

The raw data supporting the conclusions of this article will be made available by the authors, without undue reservation.

## Author Contributions

DX and NC conceived the study. NC, ZT, and XZ performed experiments, wrote the manuscripts, and revised it. BL, WL, and YT collected clinical samples. All authors have read and approved the final version of the manuscript.

## Funding

This study was supported by Jiangsu Agriculture Science and Technology Innovation Fund (JASTIF) (CX[21]3174), Forestry Science and Technology Innovation and Promotion Project of Jiangsu Province (LYKJ[2018]22 and LYKJ[2021]40), the Fundamental Research Funds for the Central Universities (KJQN202136), the Fellowship of China Postdoctoral Science Foundation (2020M681650), and the Priority Academic Program Development of Jiangsu Higher Education Institutions.

## Conflict of Interest

The authors declare that the research was conducted in the absence of any commercial or financial relationships that could be construed as a potential conflict of interest.

## Publisher's Note

All claims expressed in this article are solely those of the authors and do not necessarily represent those of their affiliated organizations, or those of the publisher, the editors and the reviewers. Any product that may be evaluated in this article, or claim that may be made by its manufacturer, is not guaranteed or endorsed by the publisher.
