# Supplementary material for: Development and Application of a Triplex TaqMan Quantitative Real-Time PCR Assay for Simultaneous Detection of Feline Calicivirus, Feline Parvovirus, and Feline Herpesvirus 1
Source: Front Vet Sci. 2022 Feb 8;8:792322. doi: 10.3389/fvets.2021.792322 (PMC8861203; doi:10.3389/fvets.2021.792322)
Supplement: Supplementary file 1 [file Data_Sheet_1.docx]

Supplementary Material

# Supplementary Tables

**Supplementary Table 1.** The quantitative real-time PCR data of Figure 1.

| **Run Order** | **Annealing Temperature (℃)** | **Primer Concentration (μM)** | **Probe Concentration (μM)** | **Ct Values** |
| --- | --- | --- | --- | --- |
| 1 | 53.00 | 0.60 | 0.05 | 14.00 |
| 2 | 60.00 | 0.60 | 0.05 | 14.60 |
| 3 | 56.50 | 0.67 | 0.13 | 14.65 |
| 4 | 53.00 | 0.60 | 0.20 | 14.70 |
| 5 | 56.50 | 0.40 | 0.13 | 14.70 |
| 6 | 51.80 | 0.40 | 0.13 | 14.85 |
| 7 | 56.50 | 0.40 | 0.23 | 14.90 |
| 8 | 56.50 | 0.40 | 0.02 | 14.95 |
| 9 | 60.00 | 0.60 | 0.20 | 15.75 |
| 10 | 53.00 | 0.20 | 0.05 | 16.00 |
| 11 | 60.00 | 0.20 | 0.05 | 16.01 |
| 12 | 60.00 | 0.02 | 0.05 | 16.25 |
| 13 | 53.00 | 0.20 | 0.20 | 16.30 |
| 14 | 53.00 | 0.20 | 0.20 | 16.40 |
| 15 | 60.00 | 0.20 | 0.20 | 17.50 |
| 16 | 56.50 | 0.13 | 0.13 | 18.00 |

**Supplementary Table 2.** Intra- and inter-assay reproducibility of the triplex assay using virus nucleic acids as templates.

| **Name** | **Virus Titer (TCID**50/assay) | **Intra-assay** a | | | **Intra-assay** a | | |
| --- | --- | --- | --- | --- | --- | --- | --- |
|  |  | **Mean** | **SD** | **CV (%)** | **Mean** | **SD** | **CV (%)** |
| FPV | 105 | 14.02 | 0.16 | 1.14 | 14.21 | 0.19 | 1.34 |
|  | 100 | 27.02 | 0.06 | 0.22 | 27.06 | 0.42 | 1.55 |
| FCV | 105 | 33.61 | 0.43 | 1.28 | 32.88 | 0.86 | 2.62 |
|  | 100 | 33.78 | 0.28 | 0.83 | 33.82 | 0.25 | 0.74 |
| FHV-1 | 105 | 23.05 | 0.25 | 1.08 | 23.53 | 0.84 | 3.57 |
|  | 100 | 34.51 | 0.59 | 1.71 | 34.59 | 0.45 | 1.30 |

^a^ Bold refers the maximum and minimum value

# Supplementary Figures

#
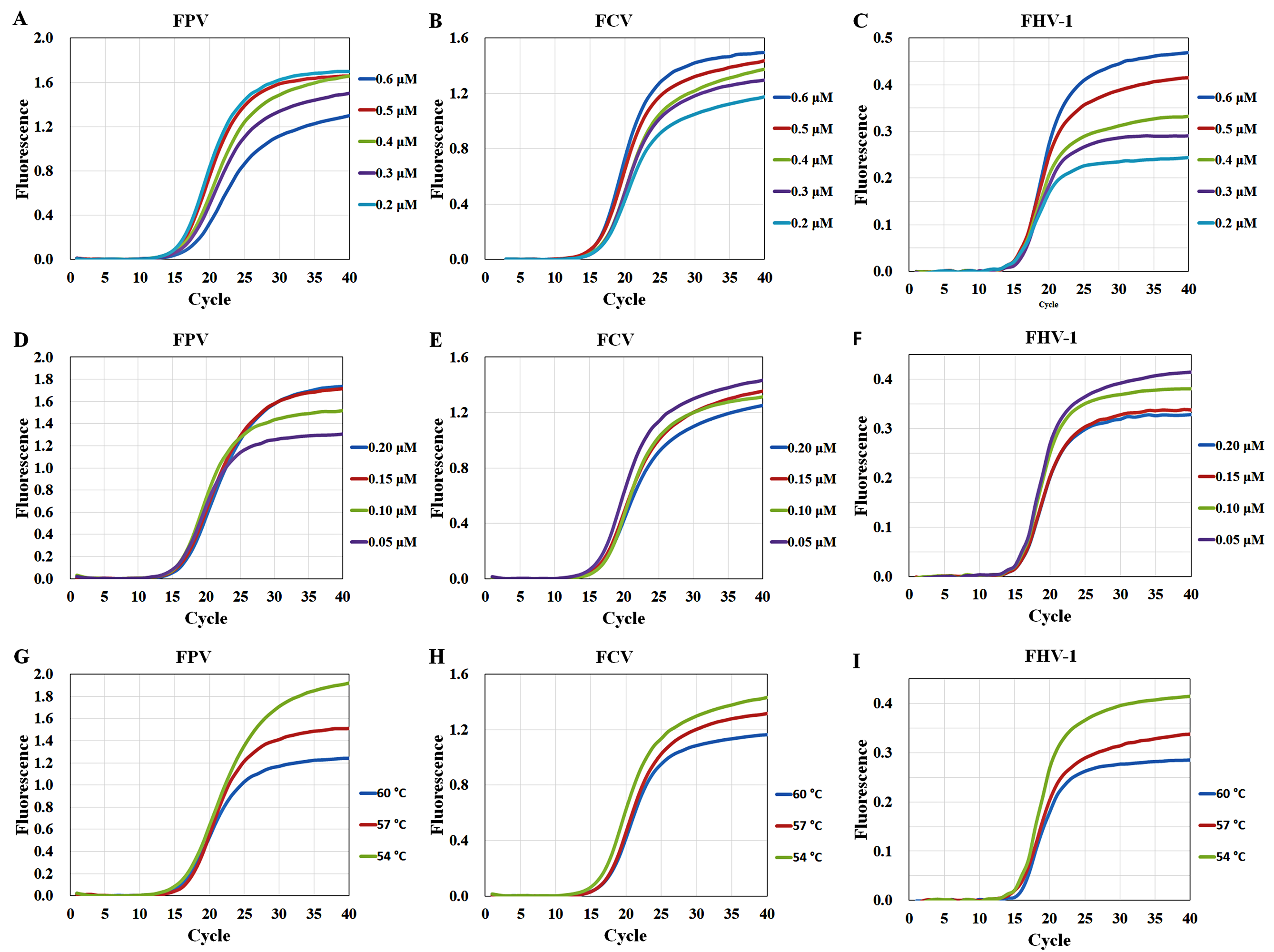


# **Supplementary Figure 1.** Effect of reaction conditions on fluorescence intensity. Effect of different primer concentrations (A, B, C), probe concentrations (D, E, F), and annealing temperatures (G, H, I) on fluorescence signal.


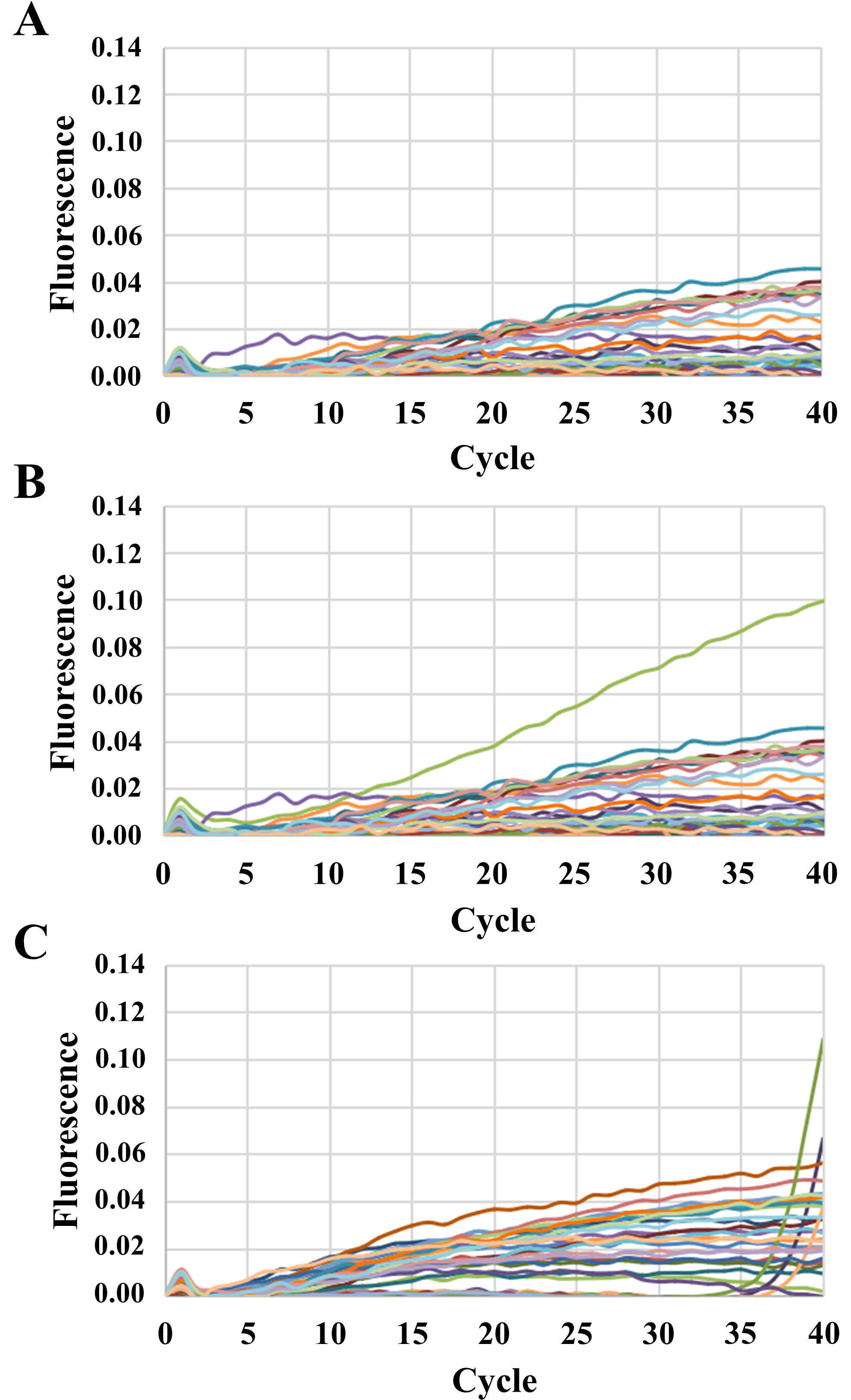


# **Supplementary Figure 2.** The amplification curves of FCV (A), FPV (B), and FHV-1 (C) plasmids at 5×10^1^ copies/assay with 30 repeats.


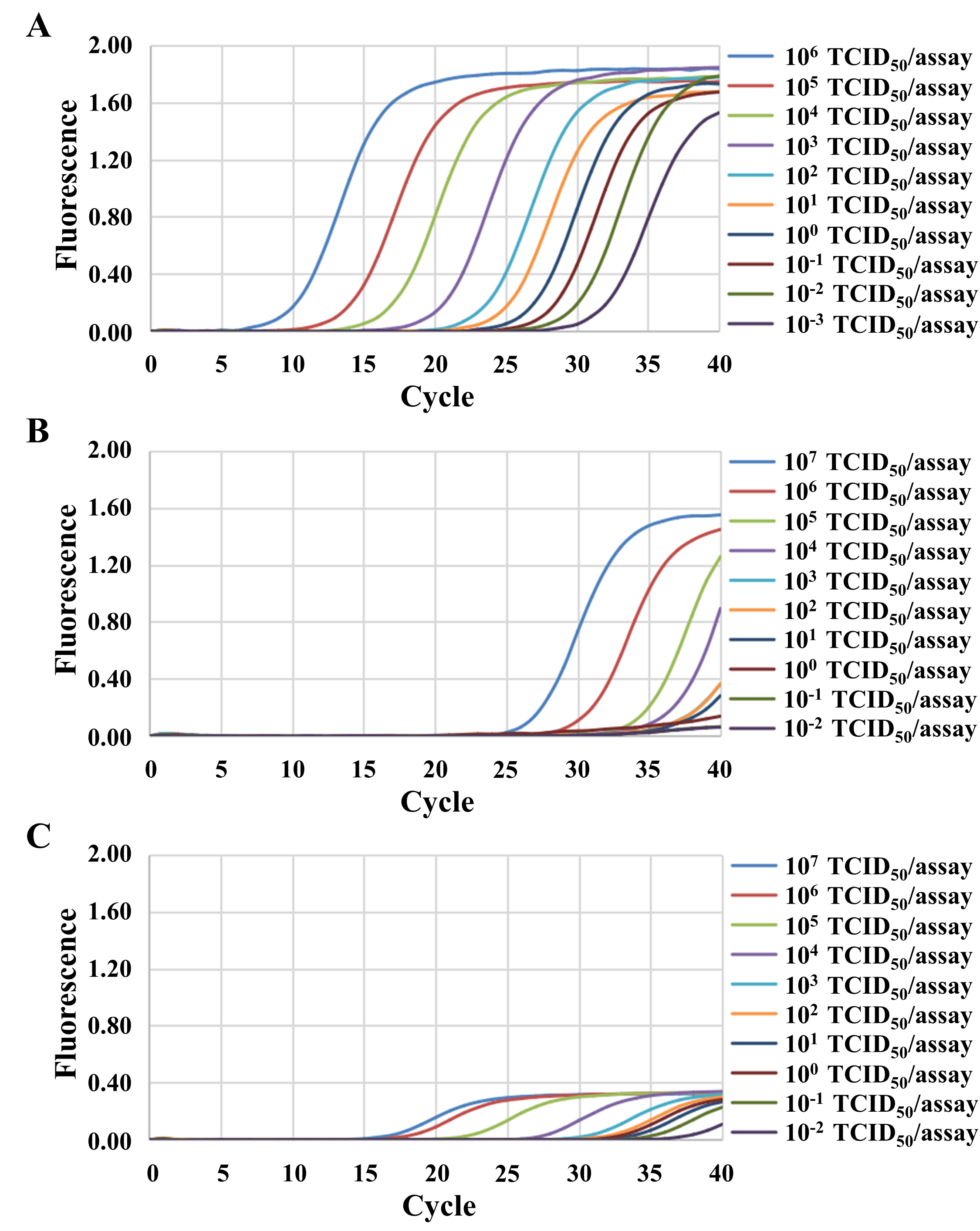


# **Supplementary Figure 3.** The amplification curves of FPV (A), FCV (B), and FHV-1 (C) nucleic acids.
